# Remnant cholesterol can identify individuals at higher risk of metabolic syndrome in the general population

**DOI:** 10.1038/s41598-023-33276-y

**Published:** 2023-04-12

**Authors:** Yang Zou, Maobin Kuang, Yanjia Zhong, Chunyuan Jiang

**Affiliations:** 1 Jiangxi Cardiovascular Research Institute, Jiangxi Provincial People’s Hospital, The First Affiliated Hospital of Nanchang Medical College, Medical College of Nanchang University, Nanchang, 330006 Jiangxi Provincial China; 2 Department of Cardiology, Jiangxi Provincial People’s Hospital, The First Affiliated Hospital of Nanchang Medical College, Nanchang, 330006 Jiangxi Provincial China

**Keywords:** Biomarkers, Medical research, Risk factors

## Abstract

Remnant cholesterol (RC) is a highly atherogenic lipid. Previous studies have shown that RC was closely associated with many metabolism-related diseases. However, the relationship of RC with metabolic syndrome (MetS) remains unclear. This study’s objective is to investigate the relationship of RC with MetS. A total of 60,799 adults who received health assessments were included in this study. RC was calculated by subtracting the directly measured values for low-density lipoprotein cholesterol (LDL-C) and high-density lipoprotein cholesterol (HDL-C) from total cholesterol (TC) and divided into 5 groups according to its quintile. MetS diagnosis according to National Cholesterol Education Program Adult Treatment Panel III (NCEP-ATP III) definitions. Application of receiver operating characteristic (ROC) curve analysis and multivariate logistic regression to assess the association of RC with MetS. In RC quintile groups, the prevalence of MetS was 0.84, 1.10, 1.92, 3.87 and 37.71%, respectively. Multivariate logical regression analysis showed that RC and MetS maintained a stable independent positive correlation between both sexes. An interaction test further showed that the MetS risk associated with RC was significantly higher in women than in men. Moreover, ROC analysis results showed that RC had high accuracy in identifying MetS, especially among young and middle-aged men [(area under the curve: AUC) < 30 years: 0.9572, 30–39 years: 0.9306, 40–49 years: 0.9067]. The current study provided the first evidence of a positive association between RC and MetS, and that this correlation was stronger in women than in man, which may be due to the relative deficiency of estrogen in women.

## Introduction

MetS is a metabolic disorder that includes a variety of adverse metabolic characteristics, such as hyperglycemia, central obesity, hypertension, and atherosclerotic lipid abnormalities^1,2^. Compared with people without these adverse metabolic characteristics, patients with MetS had a significantly higher risk of cardiovascular disease and type 2 diabetes, and further increased with age^1–4^. Epidemiological investigation and analysis from many countries showed that the global prevalence rate of MetS was about 31%, which increased the risk of coronary heart disease and cerebrovascular disease by 2 times, 1.5 times the risk of all-cause death^5^. Moreover, MetS is also strongly associated with obstructive sleep apnea, polycystic ovary syndrome, liver disease, hypogonadism, chronic kidney disease, and microvascular disease^6–8^.

RC is a highly atherogenic lipid, which includes a variety of lipoproteins such as chylomicron remnants, intermediate-density lipoprotein (IDL) and very-low-density lipoprotein (VLDL) in fasting state, also known as triglyceride-rich lipoprotein cholesterol^9,10^. In recent years, many epidemiological analyses have found that in addition to participating in and mediating the residual risk of cardiovascular disease^11,12^, RC can also be a good assessment of nonalcoholic fatty liver disease, diabetes, hypertension, chronic kidney disease, and a variety of arteriosclerosis-related diseases^13–18^. The importance of controlling the level of RC was further emphasized in the recent JACC focus discussion^19^. MetS is a metabolic disease characterized by atherosclerotic lipid abnormalities, and several previous studies have observed that MetS patients generally have higher levels of RC^20,21^. However, it is not clear about the correlation of RC levels with MetS. This study's objective is to investigate the relationship of RC with MetS, evaluate the accuracy of RC used to identify MetS, and provide new ideas for the early prevention of MetS risk.

## Material and methods

### Research data and population

This study is a post-hoc analysis of a cross-sectional study (2012–2016) of 60,799 adult participants undergoing health assessments in the Balearic Islands. This cross-sectional data was created by Romero-Saldaña et al. to validate the diagnostic performance of different non-invasive methods for MetS^22^. They invited 69,581 local employees from different economic sectors to participate in the study, of which 8,782 refused the project invitation, while the remaining 60,799 employees participated in the initial study after signing the informed consent form with a clear research purpose. In this cross-sectional dataset, the general information (age and sex) of the participants, general measurement data [diastolic blood pressure (DBP), waist circumference (WC), systolic blood pressure (SBP) and body mass index (BMI)], obesity-related parameters [a body shape index (ABSI), percentage of body fat (%BF) and waist-to-height ratio (WHtR)], tobacco consumption (smoker) and some biochemical parameters [LDL-C, TC, fasting plasma glucose (FPG), triglyceride (TG) and HDL-C] were collected. Among them, anthropometric parameter measurements were implemented in accordance with the recommendations in the "International standards for anthropometric assessment" by trained staff who took three measurements and recorded the average value^23^. Blood samples for biochemical analysis were taken from the anterior cubital vein after 12 h of fasting and then measured using automated analytical instruments according to standard procedures. Available data from the current study have been shared in a public database (Dryad database) by Professor Romero-Saldaña^24^. To make the best use of the data, the Dryad database allows a variety of researchers to use Dryad datasets for post-hoc analysis for different research purposes without infringing authors' rights.

The current study calculated RC as a lipid variable on the basis of this cross-sectional data (RC = TC − HDL-C − LDL-C)^25^, to further explore the relationship between RC and MetS. The Jiangxi Provincial People's Hospital’s Ethics Committee reviewed and approved the current research and design plan. Additionally, due to the anonymity of the dataset used in the current study, the Jiangxi Provincial People's Hospital’s Ethics Committee waived the informed consent of the participants (Review number: 2022-005). The whole study process followed the Helsinki Declaration.

### Diagnosis of MetS

In this study, the diagnosis of MetS referred to the standard of the NCEP-ATP III^26^, which can be diagnosed by containing the following three or more adverse metabolic characteristics: (1) FPG ≥ 100 mg/dL; (2) Men WC ≥ 102 cm or women WC ≥ 88 cm; (3) TG ≥ 150 mg/dL; (4) HDL-C < 50 mg/dL in women or < 40 mg/dL in men; and (5) SBP ≥ 130 mmHg and DBP ≥ 85 mmHg.

### Statistical analysis

To observe trends in the association between RC and MetS, we divided the study population into five groups according to the quintile of RC levels. Additionally, considering the obvious sex difference in the prevalence of MetS^6^, the current study discussed the association of RC with MetS for men and women separately. Three multivariate logistic regression models were constructed to calculate odds ratios (OR) and 95% confidence intervals (CI) for the association between RC and its quintiles and MetS. Before establishing the models, we analyzed the correlation between RC and MetS components through linear regression analysis; in addition, using the linear regression equation we also evaluated the collinearity of RC and covariates, calculated the variance inflation factor of RC and each covariate (Supplementary Table 1)^27^, and finally, excluded age, %BF, BMI, WHtR, TC and TG from the multivariate logistic regression models. Smoker and WC were adjusted in model 1; model 2 further adjusted ABSI, DBP, and SBP; in model 3, the blood glucose and lipid variables (HDL-C, LDL-C, and FPG) were further adjusted. In addition, to further verify whether there was a significant sex difference in the association between RC and MetS, we used Q1 of female RC as the reference group, calculated the OR and 95% CI of other RC groups in women and men, and examined sex differences in MetS risk using likelihood ratio test.

We also constructed a ROC curve to test the relative diagnostic strength of RC against MetS and calculated the AUC and best threshold of RC, and the comparison of the AUC between sexs was performed by the DeLong test^28^.

All analyses in the current study were analyzed using R language (version 3.4.3) and Empower Stats (R) (version 2.0). Baseline information were summarized as median (interquartile range) or mean (standard deviation) or percentage, respectively, depending on data type and distribution pattern. The differences between groups were identified by t-test, Kruskal–Wallis H test or Chi-square test, or one-way ANOVA. All *P* were bilateral, and *P* < 0.05 was the significance standard.

### Ethics approval and consent to participate

The Ethics Committee of Jiangxi Provincial People's Hospital reviewed and approved the current research and design plan. Additionally, due to the anonymity of the dataset used in the current study, the Ethics Committee of Jiangxi Provincial People’s Hospital abandoned the informed consent of the participants (Review number: 2022-005). The whole study process followed the Helsinki Declaration.

## Results

### Baseline characteristics of the study population

The current study included 34,827 (57.28%) men and 25,972 (42.72%) women participants with an average age of 39.98 years. According to the NCEP-ATP III diagnostic criteria, 5487 (9.02%) people were diagnosed with MetS, of which 6.74% were men and 2.28% were women.

Baseline characteristics of the research population in the RC quintile groups are shown in Table 1 (Q1: ≤ 12.38; Q2: 12.40–15.98; Q3: 16.00–20.38; Q4: 20.40–27.98; Q5: ≥ 28). There were significant differences in sex, age, %BF, HDL-C, WC, LDL-C, DBP, WHtR, SBP, ABSI, TC, TG, BMI, FPG, the number of smokers, and the prevalence of MetS among RC groups (all *P* < 0.001). Among them, the prevalence rate of MetS in the highest RC quintile (Q5) was 37.71%, which was much higher than that in other RC quintiles (Q1:0.84%, Q2:1.10%, Q3:1.92%, Q4:3.87%). Furthermore, it is worth noting that there were about four times as many men in the Q5 group as women (79.91% vs. 20.09%).

**Table 1:** Baseline characteristics according to quintiles of remnant cholesterol.

	RC quintile					*P* value
	Q1 (1–12.18)	Q2 (12.20–15.98)	Q3 (16.00–20.18)	Q4 (20.20–27.36)	Q5 (27.46–189.12)	
No. of participants	11,522	12,387	11,999	12,040	12,215	
Sex						< 0.001
Women	6652 (57.73%)	6702 (54.11%)	5723 (47.70%)	4251 (35.31%)	2601 (21.29%)	
Men	4870 (42.27%)	5685 (45.89%)	6276 (52.30%)	7789 (64.69%)	9614 (78.71%)	
Age, years	36.00 (29.00–43.00)	37.00 (30.00–45.93)	39.00 (31.00–47.00)	42.00 (34.00–50.00)	44.00 (36.00–51.00)	< 0.001
%BF	27.45 (22.19–31.93)	27.86 (23.05–32.93)	28.30 (23.40–33.80)	28.60 (23.85–34.41)	29.20 (25.00–34.45)	< 0.001
ABSI	0.07 (0.01)	0.07 (0.01)	0.07 (0.01)	0.07 (0.01)	0.07 (0.01)	< 0.001
BMI, kg/m^2^	24.05 (3.71)	24.91 (4.21)	25.79 (4.56)	26.94 (4.60)	28.48 (4.57)	< 0.001
WC, cm	78.15 (9.62)	79.65 (10.31)	81.30 (10.74)	84.29 (10.72)	90.21 (12.21)	< 0.001
WHtR	0.47 (0.05)	0.48 (0.05)	0.48 (0.06)	0.50 (0.06)	0.53 (0.07)	< 0.001
SBP, mmHg	116.01 (14.48)	117.56 (15.11)	119.14 (15.84)	123.13 (16.48)	127.12 (16.78)	< 0.001
DBP, mmHg	69.95 (9.89)	71.52 (10.23)	72.83 (10.73)	75.14 (10.90)	78.11 (11.16)	< 0.001
TC, mg/dL	173.00 (152.00–196.00)	184.00 (161.65–206.00)	191.00 (169.00–212.15)	204.00 (181.00–226.00)	215.00 (192.00–241.00)	< 0.001
HDL-C, mg/dL	54.00 (50.00–59.00)	54.00 (50.00–59.00)	54.00 (48.00–58.00)	51.00 (47.00–56.00)	48.00 (43.00–54.00)	< 0.001
LDL-C, mg/dL	107.80 (86.60–131.67)	114.60 (91.40–139.60)	119.80 (96.00–142.80)	129.65 (105.20–151.60)	128.40 (102.40–155.40)	< 0.001
TG, mg/dL	50.00 (43.00–55.00)	70.00 (66.00–75.00)	89.00 (84.00–94.00)	116.00 (108.00–125.00)	177.00 (153.00–222.00)	< 0.001
Non-HDL-C	118.00 (96.00–142.00)	129.00 (105.00–154.00)	137.00 (114.00–161.00)	153.00 (128.00–175.00)	167.00 (142.00–195.00)	< 0.001
FPG, mg/dL	84.00 (76.00–92.00)	84.00 (77.00–92.00)	85.30 (79.00–93.00)	87.00 (80.00–96.00)	90.00 (82.00–99.00)	< 0.001
Smoker (yes)	3480 (30.20%)	4092 (33.03%)	4120 (34.34%)	4288 (35.61%)	4855 (39.75%)	< 0.001
MetS (yes)	92 (0.80%)	139 (1.12%)	224 (1.87%)	389 (3.23%)	4170 (34.14%)	< 0.001

*MetS* metabolic syndrome, *SBP* systolic blood pressure, *DBP* diastolic blood pressure, *%BF* percentage of body fat, *ABSI* a body shape index, *BMI* body mass index, *WC* waist circumference, *WHtR* waist-to-height ratio, *TC* total cholesterol, *HDL-C* high-density lipoprotein cholesterol, *LDL-C* low-density lipoprotein cholesterol, *FPG* fasting plasma glucose.

Table 2 summarizes the baseline characteristics of men and women based on whether or not participants had MetS. For both sexes, MetS patients were generally older, with significantly higher levels of %BF, DBP, LDL-C, BMI, TG, WC, FPG, WHtR, ABSI, TC, and SBP. Moreover, this study also found that female smokers were relatively less likely to become MetS than non-smokers (29.35% vs. 32.64%), while the opposite was true in men (42.18% vs. 35.85%).

**Table 2:** Baseline characteristics of the MetS and non-MetS groups.

	Men			Women		
	Non-MetS	MetS	*P* value	Non-MetS	MetS	*P* value
No. of participants	30,588 (89.3%)	3646 (10.7%)		24,582 (94.6%)	1390 (5.4%)	
Age, years	39.00 (32.00–47.00)	48.00 (41.00–54.00)	< 0.001	38.36 (31.00–46.00)	49.00 (42.00–55.00)	< 0.001
%BF	24.19 (20.54–28.14)	31.03 (27.63–34.75)	< 0.001	32.27 (28.50–36.92)	42.54 (38.65–47.47)	< 0.001
ABSI	0.07 (0.01)	0.08 (0.01)	< 0.001	0.07 (0.01)	0.07 (0.01)	< 0.001
BMI, kg/m^2^	26.37 (3.92)	30.72 (4.60)	< 0.001	24.66 (4.58)	31.44 (6.00)	< 0.001
WC, cm	86.98 (8.20)	99.95 (11.53)	< 0.001	74.58 (8.91)	89.60 (13.44)	< 0.001
WHtR	0.50 (0.05)	0.58 (0.06)	< 0.001	0.46 (0.06)	0.56 (0.08)	< 0.001
SBP, mmHg	123.68 (14.79)	138.15 (16.45)	< 0.001	113.55 (14.27)	132.99 (17.60)	< 0.001
DBP, mmHg	74.91 (10.35)	84.25 (10.91)	< 0.001	69.77 (9.99)	82.08 (10.83)	< 0.001
TC, mg/dL	192.00 (168.00–216.00)	218.30 (194.00–246.00)	< 0.001	189.00 (166.00–214.00)	219.00 (196.00–246.00)	< 0.001
HDL-C, mg/dL	52.00 (47.00–55.32)	43.00 (36.70–50.00)	< 0.001	55.00 (50.00–60.00)	46.00 (43.00–50.00)	< 0.001
LDL-C, mg/dL	119.80 (95.80–143.80)	132.60 (104.40–159.60)	< 0.001	117.80 (93.20–143.00)	140.60 (116.60–166.65)	< 0.001
TG, mg/dL	96.00 (70.00–128.00)	206.00 (165.00–267.00)	< 0.001	77.00 (59.00–98.00)	161.00 (107.75–202.00)	< 0.001
Non-HDL-C, mg/dL	141.00 (115.00–166.00)	174.90 (148.00–204.00)		134.00 (108.00–161.00)	172.00 (148.00–200.00)	< 0.001
RC, mg/dL	19.20 (14.00–25.60)	41.20 (33.00–53.20)	< 0.001	15.40 (11.80–19.60)	32.20 (21.40–40.40)	< 0.001
FPG, mg/dL	87.00 (79.00–95.00)	101.00 (89.00–111.00)	< 0.001	83.00 (77.00–90.50)	100.00 (88.00–110.00)	< 0.001
Smoker (yes)	10,948 (35.79%)	1474 (40.43%)	< 0.001	8012 (32.62%)	401 (29.31%)	0.011

Abbreviations as in Table 1.

Table 3 shows the baseline characteristics of men and women in different age groups. In both sexes, compared with the younger group, the older group had larger WC, BMI, %BF, WHtR, higher incidence of MetS, and the metabolism of blood glucose, blood pressure and blood lipids were even worse. Also, it should be noted that older women had significantly higher %BF and poorer blood lipid metabolism than men and younger women.

**Table 3:** Baseline characteristics of men and women between different age groups.

	Age groups (years)					*P* value
	< 30	30–39	40–49	50–59	≥ 60	
Men						
No. of participants	5868	10,849	10,169	6184	1164	
%BF	18.92 (16.21–21.97)	22.76 (20.14–25.97)	26.27 (23.46–29.51)	29.41 (26.48–32.73)	31.97 (29.01–35.14)	< 0.001
ABSI	0.08 (0.01)	0.08 (0.01)	0.08 (0.01)	0.08 (0.01)	0.07 (0.01)	< 0.001
BMI, kg/m^2^	24.94 (4.02)	26.43 (4.08)	27.41 (4.13)	28.07 (4.07)	28.61 (3.91)	< 0.001
WC, cm	85.20 (8.53)	87.73 (9.23)	89.56 (9.59)	90.17 (9.85)	90.12 (9.36)	< 0.001
WHtR	0.49 (0.05)	0.50 (0.05)	0.52 (0.05)	0.53 (0.06)	0.53 (0.06)	< 0.001
SBP, mmHg	119.94 (12.93)	121.58 (13.27)	126.33 (14.92)	132.62 (17.96)	136.75 (18.52)	< 0.001
DBP, mmHg	70.33 (9.36)	73.84 (9.97)	78.02 (10.51)	80.53 (10.88)	80.20 (10.17)	< 0.001
TC, mg/dL	168.00 (148.00–191.00)	190.00 (168.00–213.00)	205.00 (182.00–229.00)	208.00 (185.00–231.00)	203.00 (180.00–224.00)	< 0.001
HDL-C, mg/dL	54.00 (51.00–58.00)	52.00 (48.00–56.00)	51.00 (46.00–54.00)	47.00 (43.00–51.00)	45.00 (41.00–49.00)	< 0.001
LDL-C, mg/dL	95.60 (76.00–119.00)	116.40 (94.80–139.20)	129.80 (107.20–153.00)	134.40 (111.40–156.80)	132.35 (106.20–153.20)	< 0.001
TG, mg/dL	81.00 (60.00–108.00)	97.00 (70.00–134.00)	113.00 (80.00–157.00)	119.00 (85.00–163.00)	121.00 (90.00–163.00)	< 0.001
Non-HDL-C, mg/dL	114.00 (93.00–138.00)	138.00 (115.00–162.00)	155.00 (131.00–180.00)	161.00 (137.00–185.00)	157.95 (133.00–179.00)	< 0.001
FPG, mg/dL	83.05 (76.00–91.00)	86.00 (79.00–94.00)	89.00 (81.00–97.00)	93.00 (85.00–102.00)	97.00 (87.00–109.00)	< 0.001
Smoker (yes)	2560 (43.32%)	4138 (37.59%)	3640 (35.03%)	2124 (33.51%)	284 (24.13%)	< 0.001
MetS (yes)	141 (2.40%)	650 (5.99%)	1314 (12.92%)	1246 (20.15%)	295 (25.34%)	< 0.001
Women						
No. of participants	4988	8404	7819	4072	646	
%BF	27.41 (24.88–30.99)	30.51 (27.75–34.37)	34.34 (31.34–38.34)	38.11 (34.85–42.16)	40.87 (38.01–44.86)	< 0.001
ABSI	0.07 (0.01)	0.07 (0.01)	0.07 (0.01)	0.07 (0.01)	0.07 (0.01)	< 0.001
BMI, kg/m^2^	23.48 (4.60)	24.40 (4.87)	25.58 (4.82)	26.75 (4.75)	27.42 (4.53)	< 0.001
WC, cm	72.84 (8.70)	74.33 (9.34)	76.65 (10.29)	77.80 (10.00)	77.75 (9.85)	< 0.001
WHtR	0.45 (0.05)	0.46 (0.06)	0.48 (0.06)	0.49 (0.06)	0.50 (0.06)	< 0.001
SBP, mmHg	109.05 (11.61)	110.63 (12.70)	116.60 (14.99)	123.43 (17.03)	128.37 (17.24)	< 0.001
DBP, mmHg	66.45 (8.66)	68.26 (9.56)	72.13 (10.42)	75.52 (10.80)	76.44 (10.38)	< 0.001
TC, mg/dL	171.00 (152.00–194.00)	182.40 (161.00–205.00)	197.00 (176.00–219.05)	215.00 (194.00–238.00)	220.00 (197.62–239.75)	< 0.001
HDL-C, mg/dL	57.00 (52.00–63.00)	55.00 (50.00–60.00)	54.00 (48.00–59.00)	51.00 (46.00–57.00)	50.00 (45.00–56.00)	< 0.001
LDL-C, mg/dL	98.00 (77.35–121.60)	110.80 (88.60–133.80)	125.78 (103.96–148.00)	143.40 (121.00–165.80)	145.22 (123.85–167.60)	< 0.001
TG, mg/dL	74.00 (57.00–92.00)	75.00 (57.00–95.00)	80.00 (61.00–104.00)	90.00 (67.00–122.00)	102.00 (73.47–132.00)	< 0.001
Non-HDL-C, mg/dL	113.00 (92.00–138.00)	126.00 (103.00–152.00)	143.00 (119.00–167.00)	163.00 (140.00–187.00)	169.00 (144.00–190.00)	< 0.001
FPG, mg/dL	81.00 (74.00–88.00)	83.00 (76.00–90.00)	85.00 (78.00–92.00)	88.00 (81.00–96.00)	90.00 (82.00–101.00)	< 0.001
Smoker (yes)	1844 (36.97%)	2659 (31.64%)	2691 (34.42%)	1139 (27.97%)	80 (12.38%)	< 0.001
MetS (yes)	46 (0.92%)	206 (2.45%)	457 (5.84%)	520 (12.77%)	139 (21.52%)	< 0.001

Abbreviations as in Table 1.

### Association of RC with MetS components

By linear regression analysis, we assessed the association of RC with WC, SBP, DBP, HDL-C, TG, and FPG (Supplementary Table 2). The results of the study showed that RC was associated with each of the components of the MetS, with the highest magnitude of association with WC (β = 0.49, 95%CI:0.48–0.49) and HDL-C (β = − 0.48, 95%CI: − 0.49–0.47); these results suggested that RC may be a risk factor for MetS.

### Association between RC and risk of MetS

The results of the multivariate logistic regression analysis of RC and MetS are shown in Table 4. From Model 1 to Model 3, RC and MetS were positively correlated in both sexes, and the degree of correlation sustained stable. From the results of the association analysis between the RC quintile and MetS, we also found that the risk of MetS gradually increased as the quintiles of RC elevated (*P*-trend < 0.0001), and higher quintiles of RC (Q5) suggested a very high MetS risk [Model 3: OR = 33.28 (23.02, 48.11) for men, OR = 23.39 (17.17, 31.87) for women]. Additionally, to further verify sex differences in the association of RC and MetS between men and women, we used Q1 of female RC as the reference group to calculate the OR and 95% CI of other RC groups in women and men (Table 5). The results showed that men had a significantly lower risk of RC-related MetS compared to women. In order to verify whether the sex difference in the association between RC and MetS was caused by different standards for the use of HDL-C, we continued to conduct two sensitivity analyses. In these two analyses, we unified the HDL-C criteria for both sexes in the diagnosis of MetS, in which Sensitivity-1 used HDL-C < 40 mg/dL in both sexes as one of the criteria for the diagnosis of MetS in both sexes, while Sensitivity-2 setting this value to HDL-C < 50 mg/dL. It is worth noting that, following the harmonization of diagnostic criteria for HDL-C in MetS, the results of the sensitivity analyses were similar to the previous results in that there were gender differences in the relationship between RC and MetS (Supplementary Table 3), with the risk of MetS associated with RC being stronger in women than in men.

**Table 4:** Logistic regression analyses for the association between RC and MetS in different models.

	Odds ratios (95% confidence interval)			
	Crude model	Model 1	Model 2	Model 3
Men				
RC (continuous variable)				
	1.12 (1.12, 1.13)	1.10 (1.10, 1.11)	1.11 (1.11, 1.12)	1.11 (1.11, 1.12)
RC quintile				
Q1	Ref	Ref	Ref	Ref
Q2	1.35 (0.87, 2.10)	1.08 (0.69, 1.69)	1.00 (0.63, 1.60)	0.87 (0.53, 1.44)
Q3	1.77 (1.18, 2.67)	1.21 (0.80, 1.84)	1.05 (0.68, 1.62)	0.82 (0.51, 1.32)
Q4	3.72 (2.57, 5.40)	2.22 (1.52, 3.24)	1.73 (1.16, 2.57)	1.19 (0.77, 1.83)
Q5	76.36 (54.09, 107.81)	39.10 (27.55, 55.50)	40.37 (27.99, 58.22)	32.38 (21.71, 48.30)
*P*-trend	< 0.0001	< 0.0001	< 0.0001	< 0.0001
Women				
RC (continuous variable)				
	1.15 (1.14, 1.15)	1.13 (1.13, 1.14)	1.13 (1.13, 1.14)	1.13 (1.12, 1.14)
RC quintile				
Q1	Ref	Ref	Ref	Ref
Q2	1.47 (1.05, 2.05)	1.25 (0.89, 1.76)	1.03 (0.72, 1.47)	0.93 (0.64, 1.36)
Q3	2.99 (2.20, 4.05)	2.22 (1.62, 3.04)	1.61 (1.16, 2.24)	1.21 (0.85, 1.73)
Q4	5.40 (4.03, 7.24)	3.46 (2.55, 4.69)	1.99 (1.44, 2.75)	1.17 (0.83, 1.66)
Q5	56.85 (43.44, 74.38)	37.10 (28.13, 48.94)	29.39 (21.96, 39.33)	20.70 (15.11, 28.36)
*P*-trend	< 0.0001	< 0.0001	< 0.0001	< 0.0001

Abbreviations as in Table 1.

Model 1 adjusted for smoke and WC;

Model 2 adjusted for smoke, WC, ABSI, SBP and DBP;

Model 3 adjusted for smoke, WC, ABSI, SBP, DBP, HDL-C, LDL-C and FPG.

**Table 5:** Effect of RC on MetS risk stratified by sex.

	RC quintile					*P*-interaction
	Q1	Q2	Q3	Q4	Q5	
Sex						< 0.0001
Women	Ref	0.93 (0.64, 1.36)	1.21 (0.85, 1.73)	1.17 (0.83, 1.66)	20.70 (15.11, 28.36)	
Men	0.29 (0.06, 1.48)	0.26 (0.05, 1.27)	0.24 (0.05, 1.19)	0.35 (0.07, 1.70)	9.52 (2.01, 45.11)	

Abbreviations as in Table1.

Adjusted for smoke, WC, ABSI, SBP, DBP, HDL-C, LDL-C and FPG.

### Accuracy of RC for identifying MetS

**Figure 1:**
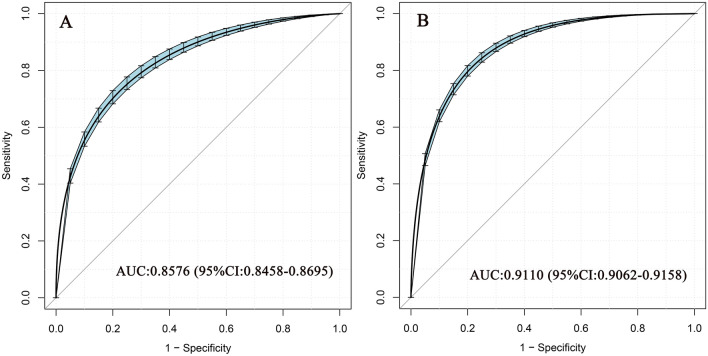
ROC analysis of RC for the identification of MetS in women (**A**) and men (**B**). ROC: receiver operating characteristic; RC: remnant cholesterol; MetS: metabolic syndrome.

ROC curve was established to evaluate the accuracy of RC in identifying MetS in both sexes. As shown in Fig. 1, RC showed high diagnostic accuracy in identifying MetS, and the AUC and best threshold for men and women were 0.9110, 0.8576 and 29.81, 23.59, respectively; Further Delong test results showed that RC was significantly more accurate in identifying MetS in men than in women (*P* < 0.001, DeLong test). Besides, we further evaluated the accuracy of RC in identifying MetS in different age groups of both sexes (Table 6). Overall, whether men or women, the accuracy of RC in identifying MetS gradually decreased with aging, which was higher in young and middle-aged people, and lowest in people aged 60 and above. In order to verify whether there was a significant difference in the accuracy of RC in identifying MetS between different age groups, we further compared the differences between people ≥ 60 years old and other younger people (< 60 years old) by the DeLong test. The results showed that the accuracy of RC in identifying MetS in people ≥ 60 years old decreased significantly compared with other younger people, both in men and women (All *P* < 0.001, DeLong test).

**Table 6:** Areas under the receiver operating characteristic curves for RC in identifying MetS in subjects of different ages.

Age, years	AUC	95%CI low	95%CI upp	Best threshold	Specificity	Sensitivity
Men						
≥ 60	0.8605	0.8352	0.8859	29.8100	0.8573	0.8034
50–59	0.8725*	0.8608	0.8842	29.9900	0.8264	0.8491
40–49	0.8996*	0.8910	0.9082	29.9700	0.8121	0.9117
30–39	0.9244*	0.9137	0.9351	29.9900	0.8566	0.9246
< 30	0.9516*	0.9347	0.9684	30.1000	0.9137	0.9362
Women						
≥ 60	0.8046	0.7591	0.8502	27.3000	0.8994	0.6475
50–59	0.8368*	0.8151	0.8584	29.9800	0.9389	0.6423
40–49	0.8358*	0.8133	0.8584	23.5900	0.8551	0.6937
30–39	0.8590*	0.8301	0.8879	28.7000	0.9533	0.6262
< 30	0.8771*	0.8177	0.9366	28.7000	0.9549	0.6957

*AUC* area under the curve, *CI* confidence interval; other abbreviations as in Table 1.

*P < 0.001, comparing the AUC of the ≥ 60 group with other age groups by Delong test.

## Discussion

In the current population-based study we found that higher RC concentrations were significantly related to increased risk of MetS. After further adjustment of other non-collinear covariates, the relationship and degree of association between RC and MetS were almost unchanged. Additionally, ROC analysis results further proved that RC had high accuracy in identifying MetS.

MetS is one of the most common chronic non-communicable diseases worldwide. Previous studies have confirmed that atherosclerotic lipid abnormalities were closely related to MetS, among which TG and HDL-C have received extensive attention in the past and become one of the diagnostic criteria of MetS^1–6^. Non-HDL-C is an atherogenic lipid that has been studied in recent years, and some recent studies have also shown that this parameter was independently positively correlated with MetS^29,30^. An analysis of national data in Iran showed that non-HDL-C had an AUC of 0.719 in identifying MetS in adults^31^. RC is the cholesterol present in triglyceride-rich lipoproteins and these lipoproteins are the direct precursors of atherogenic small dense LDL (sd-LDL) with a highly atherogenic effect^9,32^. According to the findings of Prof. Fujioka, they believed that residual lipoproteins were key particles in the formation of atherosclerosis^10^, while in a recent study by Sascău et al., they described RC as a silent promoter of metabolic diseases^33^. Some published observational studies have also confirmed that there was a significant association of RC with cardiovascular diseases and other metabolic-related diseases^11–18^, among which cardiovascular risk was considered to be caused by the direct action of VLDL/IDL precursors through lipases (lipoprotein lipase and hepatic lipase)^34–36^. Additionally, please note that, in some recent studies, researchers also found that sd-LDL seems to be an important predictor of cardio-cerebrovascular events in patients with MetS, and is directly related to MetS^37–39^. Therefore, we hypothesized that triglyceride-rich lipoprotein cholesterol RC may also be closely associated with MetS. To clarify the relationship of RC with MetS, the current study conducted a systematic analysis of 60,799 adults from different economic sectors. The results showed that a higher RC level was independently association with increased MetS risk, and RC had high accuracy in the identification of MetS. For all I know, this is the first study on the association of RC with MetS. The high accuracy of RC in identifying MetS further provides a powerful tool for risk assessment of the general population.

Notably, in the current study we also found a significant sex difference in MetS risk associated with RC. Compared with men, women have a significantly higher risk of MetS related to RC. This sex difference was odd, since the proportion of men in the highest RC group was much higher than that of women, but from the results of the interactive test, the risk of MetS related to RC in women was higher than that in men. The answer to this particular phenomenon may be inspired by the baseline characteristics of the participants. As can be seen from baseline characteristics in Table 2, women indeed had lower RC levels compared to man participants with MetS, but their age, %BF, was much higher. After further stratifying by age, we found that older women had significantly higher %BF and worse lipid metabolism than men and younger women. According to the survey, the %BF of premenopausal women in Spain was about 30%^40^, which was consistent with the results of the current study of young and middle-aged women (%BF among 19–49 years: 27.41–34.34%). Additionally, in the current study, we also found that the prevalence rate of MetS in middle-aged and elderly women was much higher than that in young and middle-aged women [Prevalence of MetS: Women: 0.94% (< 30 years) vs. 2.52% (30–39 years) vs. 5.94% (40–49 years) vs. 12.90% (50–59 years) vs. 21.64% (≥ 60 years)], among which the prevalence rate of MetS in women ≥ 50 years old was about 3.7 times higher than that in women < 50 years old, while in men, the proportion was about 1.98. In addition, according to the results of ROC analysis, we also found that the accuracy of RC in identifying MetS gradually decreased with the increase of age; compared with younger individuals, there was a significant decrease in the accuracy of RC in identifying MetS in people ≥ 60 years old. Based on the above analyses, the explanation that the risk of MetS in women was higher than that in men may be more attributed to advanced age, and from the point of view of the increasing trend of age and %BF, these changes seemed to be in line with the physical characteristics of postmenopausal women. As everyone knows that postmenopausal women experience a series of physiological changes, the most obvious of which is the redistribution of adipose tissue, especially the rapid increase in visceral fat^41,42^, which is related to the role of pro-inflammatory adipokines and biomarkers associated with atherogenic lipoproteins^43–45^. When visceral fat increases, it will enhance the decomposition of visceral fat by lipoprotein lipase in adipose tissue, which leads to the production of excessive free fatty acids, leading to insulin resistance (IR) and MetS^46,47^. Additionally, with the beginning of menopause, the decline of skeletal muscle mass and strength is accelerated^47,48^, blood lipids deteriorate, and RC levels are significantly increased^49^, all of which cause IR, which in turn leads to MetS^47,50^. In summary, the main consideration for the higher risk of MetS associated with women in the current study may be related to the relative ideficiency of estrogen in women. Estrogen replacement therapy (ERT) has long been considered to play a beneficial role in menopausal symptom management, preventing menopause-related cardiovascular disease risk, osteoporosis, MetS, vaginal epithelial thinning and hot flashes^51–53^. However, it has had a huge impact on the clinical application of ERT after the results of the Women’s Health Initiative (WHI) Hormone Trial were reported, in which ERT prescriptions were reduced by more than 50% in Spain, where up to now the ERT application rate is about 5.0–9.4%^54^. The main reason for the significant decline in the clinical application of ERT is that the results of WHI are completely contrary to previous perceptions: the results of WHI showed that ERT did not support the idea of protecting the cardiovascular system, and this adverse result may be related to the fact that WHI enrolled many unhealthy obese women and those with MetS^53,55,56^; moreover, the WHI results also indicated that ERT significantly increased the risk of breast cancer and venous thrombosis^55^. All in all, the overall benefits of postmenopausal women receiving ERT are still controversial^53,57^. Although existing studies generally support that postmenopausal women with MetS can benefit from ERT^52,58,59^, it is still necessary to pay attention to cardiovascular risks and adverse reactions of patients with MetS after ERT treatment^60^.

Also, to be mentioned that RC in this study was calculated by other lipid parameters. The accuracy of the directly calculated RC has also been discussed and analyzed in some previous high-quality studies. Generally speaking, there is no significant difference between the directly calculated RC and the measured RC^61–63^. Considering the macromolecular heterogeneity of RC, there are relatively high technical requirements for the separation and detection of residual lipoproteins^62^, which will limit their clinical application. The calculation of RC as an alternative method is very convenient and greatly saves time and economic costs. It will be of great help in disease prevention and treatment decision-making in our clinical practice.

## Study strength and limitations

The strength of this research is that the association between RC and MetS has been revealed for the first time through a strict statistical analysis strategy supported by super-large sample data.

The limitations of current research mainly come from the following aspects: (1) As mentioned earlier, the value of RC in this study was obtained by calculating. Although the previous research evidence supported the clinical application of the calculated RC^61–63^, it is undeniable that if there are measured RC data, it will further support the current research results; (2) The current study is the first study on the relationship between RC and MetS, which lacks the comparison of similar studies, and more research data from different ethnic groups are needed to verify the reliability of the conclusions; (3) The current research was a secondary analysis of the previous research dataset, in which the covariables contained in the dataset were limited, so it is inevitable that some residual confounding was not considered in the current research^64^, and further research is needed; (4) This study did not adopt a longitudinal study design, so it was not possible to assess the causal association between RC and MetS; (5) As the baseline medication use of subjects was not recorded in this larger sample cross-sectional survey, this may have led to missed diagnoses in some populations due to the use of medications for hypertension, diabetes, and dyslipidemia, reducing the prevalence of metabolic syndrome in the current study. On the flip side, however, the current study still found an association between RC and MetS at much lower prevalence rates, so, that may be considered a strong and robust association.

## Conclusion

The current study provided the first evidence of a positive association between RC and MetS, and this association was stronger in women than in men, which may be due to the relative deficiency of estrogen in women. Additionally, ROC analysis results showed that RC was a very accurate lipid parameter for identifying MetS, a finding which further suggested that RC may be a simple and economically useful marker for assessing MetS risk.

## Supplementary Information

Supplementary Information.

## Data Availability

The datasets presented in this study can be found in online repositories. The names of the repository/repositories and accession number(s) can be found below: “Romero-Saldaña, Manuel et al. (2018), Data from: Validation of a non-invasive method for the early detection of metabolic syndrome: a diagnostic accuracy test in a working population, Dryad, Dataset, https://doi.org/10.5061/dryad.cb51t54”.

## Abbreviations

RC: Remnant cholesterol
MetS: Metabolic syndrome
VLDL: Very-low-density lipoprotein
IDL: Intermediate-density lipoprotein
DBP: Diastolic blood pressure
BMI: Body mass index
SBP: Systolic blood pressure
WC: Waist circumference
WHtR: Waist-to-height ratio
%BF: Percentage of body fat
ABSI: Body shape index
HDL-C: High-density lipoprotein cholesterol
TG: Triglyceride
FPG: Fasting plasma glucose
TC: Total cholesterol
LDL-C: Low-density lipoprotein cholesterol
NCEP-ATP III: National Cholesterol Education Program Adult Treatment Panel III
OR: Odds ratio
CI: Confidence interval
ROC: Receiver operating characteristic
AUC: Area under the curve
