# Supplementary material for: Remnant cholesterol can identify individuals at higher risk of metabolic syndrome in the general population
Source: Sci Rep. 2023 Apr 12;13:5957. doi: 10.1038/s41598-023-33276-y (PMC10097632; doi:10.1038/s41598-023-33276-y)
Supplement: Supplementary file 1 — Supplementary Information. [file 41598_2023_33276_MOESM1_ESM.docx]

Supplementary Table 1: Collinearity diagnostics steps.

|  | VIF | | | | | | | |
| --- | --- | --- | --- | --- | --- | --- | --- | --- |
|  | Step 1 | Step 2 | Step 3 | Step 4 | Step 5 | Step 6 | Step 7 | Step 8 |
| RC | Inf | Inf | Inf | 340.7 | 1.3 | 1.3 | 1.3 | 1.3 |
| Age | Inf | NA | NA | NA | NA | NA | NA | NA |
| Sex | Inf | 9.2 | 9.2 | 9.2 | 9.2 | 8.6 | 8.1 | 1.5 |
| Smoker | 1 | 1 | 1 | 1 | 1 | 1 | 1 | 1 |
| %BF | Inf | 14.9 | 14.9 | 14.9 | 14.9 | 13.6 | 9.1 | NA |
| ABSI | 40.9 | 40.9 | 40.9 | 40.9 | 40.9 | 6.7 | 5 | 1.6 |
| BMI | Inf | 60 | 60 | 60 | 60 | NA | NA | NA |
| WC | 32.1 | 32.1 | 32.1 | 32.1 | 32.1 | 13.5 | 9 | 2.3 |
| WHtR | 27.8 | 27.8 | 27.8 | 27.8 | 27.8 | 14.5 | NA | NA |
| SBP | 2.5 | 2.5 | 2.5 | 2.5 | 2.5 | 2.5 | 2.4 | 2.4 |
| DBP | 2.4 | 2.4 | 2.4 | 2.4 | 2.4 | 2.4 | 2.4 | 2.4 |
| TC | Inf | Inf | NA | NA | NA | NA | NA | NA |
| HDL-C | Inf | Inf | 1.3 | 1.3 | 1.3 | 1.3 | 1.3 | 1.3 |
| Non-HDL-C | Inf | Inf | Inf | NA | NA | NA | NA | NA |
| LDL-C | Inf | Inf | Inf | 1.3 | 1.3 | 1.3 | 1.3 | 1.2 |
| TG | 340.6 | 340.6 | 340.6 | 340.6 | NA | NA | NA | NA |
| FPG | 1.1 | 1.1 | 1.1 | 1.1 | 1.1 | 1.1 | 1.1 | 1.1 |

Abbreviation: VIF: variance inflation factor; VIF = 1/(1-R^2^). Abbreviations as in Table ​1.

Note: The variables with VIF>5 will be regarded as collinear variables and cannot be included in the multiple regression model.

Supplementary Table 2: association between each component of metabolic syndrome and RC.

|  | Effect size (β), 95%CI | *P*-value |
| --- | --- | --- |
| WC | 0.49 (0.48, 0.49) | <0.0001 |
| SBP | 0.20 (0.19, 0.20) | <0.0001 |
| DBP | 0.30 (0.30, 0.31) | <0.0001 |
| HDL-C | -0.48 (-0.49, -0.47) | <0.0001 |
| TG | 0.18 (0.17, 0.18) | <0.0001 |
| FPG | 0.16 (0.16, 0.17) | <0.0001 |

Abbreviations as in Table ​1.

Supplementary Table 3: Effect of RC on MetS risk stratified by sex.

|  | RC quintile | | | | |  | |  |
| --- | --- | --- | --- | --- | --- | --- | --- | --- |
|  | Q1 | Q2 | Q3 | Q4 | Q5 | *P*-interaction | |  |
| Sensitivity-1 |  |  |  |  |  | |  | |
| Sex |  |  |  |  |  | | <0.0001 | |
| Women | Ref | 1.27 (0.64, 2.50) | 1.05 (0.55, 2.01) | 1.11 (0.60, 2.08) | 29.21 (16.63, 51.31) | |  | |
| Men | 0.21 (0.11, 0.43) | 0.13 (0.07, 0.26) | 0.16 (0.08, 0.29) | 0.28 (0.16, 0.50) | 10.43 (6.02, 18.04) | |  | |
| Sensitivity-2 |  |  |  |  |  | |  | |
| Sex |  |  |  |  |  | | 0.0065 | |
| Women | Ref | 0.97 (0.65, 1.44) | 1.05 (0.72, 1.52) | 0.98 (0.68, 1.40) | 23.77 (17.16, 32.91) | |  | |
| Men | 0.46 (0.32, 0.66) | 0.43 (0.30, 0.61) | 0.37 (0.26, 0.51) | 0.39 (0.28, 0.54) | 6.80 (5.00, 9.24) | |  | |

Abbreviations as in Table ​1.

Note 1: The diagnostic criteria for MetS in Sensitivity-1 are as follows: (1) HDL-C < 40 mg/dL; (2) Men WC ≥ 102 cm or women WC ≥ 88 cm; (3) TG ≥ 150 mg/dL; (4) FPG ≥ 100 mg/dL; and (5) SBP ≥ 130mmHg and DBP ≥ 85mmHg.

Note 2: The diagnostic criteria for MetS in Sensitivity-2 are as follows: (1) HDL-C < 50 mg/dL; (2) Men WC ≥ 102 cm or women WC ≥ 88 cm; (3) TG ≥ 150 mg/dL; (4) FPG ≥ 100 mg/dL; and (5) SBP ≥ 130mmHg and DBP ≥ 85mmHg.

Adjusted for smoke, WC, ABSI, SBP, DBP, HDL-C, LDL-C and FPG.
